# Small RNAs detected in exosomes derived from the MH7A synovial fibroblast cell line with TNF-α stimulation

**DOI:** 10.1371/journal.pone.0201851

**Published:** 2018-08-10

**Authors:** Yosuke Takamura, Wataru Aoki, Atsushi Satomura, Seiji Shibasaki, Mitsuyoshi Ueda

**Affiliations:** 1 Division of Applied Life Sciences, Graduate School of Agriculture, Kyoto University, Kitashirakawa Oiwake-cho, Sakyo-ku, Kyoto, Japan; 2 General Education Center, Hyogo University of Health Sciences, Kobe, Japan; Kunming University of Science and Technology, CHINA

## Abstract

Rheumatoid arthritis (RA) is an autoimmune disease that causes the chronic inflammation of the joints. Intercellular communication containing synovial fibroblasts seems to play a major role in RA pathogenesis. In this study, to better understand intercellular communication related to RA pathogenesis, we identified exosomal microRNAs (miRNAs) derived from synovial fibroblasts. Exosomes were collected from an RA synovial fibroblast (RASF) cell line, namely, MH7A, with or without stimulation by tumor necrosis factor alpha (TNF-α). We used small RNA sequencing to analyze the profile of small RNAs, including miRNAs, in MH7A exosomes and cells. By using differential expression analysis, we identified four miRNAs (miR-155-5p, miR-146a-5p, miR-323a-5p, and miR-1307-3p) that are upregulated in exosomes with TNF-α stimulation. The identification of miR-155-5p and miR-146a-5p which have been reported in RA patients demonstrated the validity of our experimental model. Other two miRNAs were newly identified. miR-323a-5p was predicted to target the protein encoding gene *CD6*, which attenuates T-cell activation signals, and miR-1307-3p was predicted to target the protein encoding gene N-myc downstream-regulated gene 2 (*NDRG2*), which inhibits osteoclast-related gene expression. The results suggested that these miRNAs might be involved in RA pathogenesis. We hope our results will help us understand the role of RASF exosomes in RA pathogenesis.

## Introduction

Rheumatoid arthritis (RA) is a well-known autoimmune disease characterized by chronic synovial inflammation leading to joint destruction [1]. The RA synovium comprises abnormally proliferating synovial fibroblasts, macrophages, and infiltrated leucocytes [2]. RA synovial fibroblasts (RASFs) play a major role in the formation of intercellular communication networks in the synovium via chemokines and inflammatory cytokines such as tumor necrosis factor alpha (TNF-α) and interleukin-6 (IL-6), which drive synovial inflammation and joint destruction [2–4]. However, despite advances in RA treatment with biological agents, there are still some cases of uncontrolled synovial inflammation [5].

Exosomes, a type of extracellular vesicle, also participate in intercellular communication; they play a role in transporting bioactive molecules such as proteins and various types of RNAs, including microRNAs (miRNAs) [6]. Exosomes are secreted from all types of cells and are present in all biological fluids [6]. Exosomal miRNAs show physiological and pathological functions including immune regulation and cancer progression [7, 8], and the exosome transportation is not just a dead cell clearance mechanism [9]. For example, Ochiya’s group showed that breast cancer-derived exosomes containing miR-181c trigger the breakdown of blood-brain barrier, and lead to brain metastasis [10]. There are several pathways in exosome uptake: receptor-ligand interactions, direct membrane fusion, and endocytosis/phagocytosis [11]. Studies showed that the primary pathway is internalization via micropinocytosis, clathrin-mediated and caveolin-mediated endocytosis, and phagocytosis. Many exosomes are targeted to the lysosome, but some of them escape lysosomal degradation via back fusion and release their contents into the cytoplasm [12]. Released miRNAs show their function in various ways [13]. For example, some portion of exosomal miRNAs associate with AGO2 and show the classic role of targeting mRNAs with the RISC components [14]. Furthermore exosomal miRNAs have entirely new functions as ligands for activation of immune cells via interaction with toll-like receptors [15]. In RA, exosomes derived from the synovial fluid contain citrullinated proteins [16], and RASF-derived exosomes contain a membrane form of TNF-α [17]. The molecular cargo in exosomes changes according to the origin and state of the cells, thus suggesting that exosomal proteins are involved in RA pathogenesis and that exosomes have the potential to be used as biomarkers [6, 7].

miRNAs are regulatory, noncoding RNA molecules comprising ~22 nucleotides. According to miRBase (http://www.mirbase.org/), an miRNA sequence and annotation database, more than 1800 miRNAs have been discovered in the human genome [18]. miRNAs play an important role in biological processes, including immune system regulation via interaction with partially complement messenger RNA (mRNA) to suppress mRNA translation [19, 20]. Several studies have evaluated miRNA expression in plasma, synovial tissue, and synovial fibroblasts in RA patients [21]. One study has shown that because miR-132 levels in the plasma of RA patients are different from those in healthy controls, miR-132 levels can be used as a potential biomarker [22]. Another study has shown that miR-155 and miR-146a levels increase in RASF [23]. Furthermore, miR-155-deficient mice do not develop collagen-induced arthritis (an RA model in mice), thus suggesting that miR-155 is involved in the chronic inflammation and joint destruction mechanisms [24]. Taken together, these miRNAs could contribute to RA pathogenesis and could be used as biomarkers for RA.

In addition to miRNAs, exosomes contain other types of noncoding RNAs, including ribosomal RNAs (rRNAs), transfer RNAs (tRNAs), small nuclear RNAs (snRNAs), small nucleolar RNAs (snoRNAs), long noncoding RNAs (lncRNAs), small interfering RNAs (siRNAs), PIWI-interacting RNAs (piRNAs), Y RNAs, and vault RNAs (vtRNAs) [25–30]. These RNAs are different in function, localization, length, and structure. Although many questions still remain about these noncoding RNAs, their functions have become increasingly clear [25].

**Fig 1:**
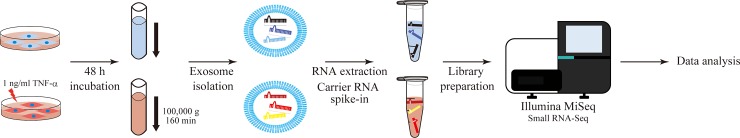
Experimental procedure of this study. Exosomes were isolated by ultracentrifugation from MH7A-conditioned media with or without TNF-α stimulation. Carrier RNA was used in the extraction step of exosomal RNAs. The extracted exosomal RNAs were sequenced on Illumina MiSeq.

In this study, we focused on RASF-derived exosomal RNAs to elucidate their role in RA pathogenesis. We performed small RNA sequencing (RNA-Seq) by using Illumina MiSeq to analyze the miRNA expression of RASF exosomes. To construct a model of RA *in vitro*, we cultured MH7A cells—a human synovial fibroblast cell line derived from intra-articular soft tissues of the knee joints of RA patients [31]. The cells were stimulated with TNF-α to produce inflammation, and the profile of the exosomal miRNA was compared with that of unstimulated MH7A cells. Furthermore, we analyzed cellular RNA profiles to compare the expression profiles between exosomes and cells. Fig 1 illustrates the experimental system of this study. We isolated MH7A exosomes from MH7A-conditioned media and quantified exosomal small RNAs. With TNF-α stimulation, four miRNAs were found to be upregulated in RASF exosomes. We believe our observations on RASF exosomal RNAs will help us understand the role of RASF exosomes in RA pathogenesis.

## Materials and methods

### Cell culture

In this study, we used the human synovial fibroblast cell line MH7A (Riken, Saitama, Japan) [4, 31]. MH7A cells were grown in a Biocoat collagen I 100 mm dish (Corning, NY, USA) with Roswell Park Memorial Institute 1640 Medium (Wako, Osaka, Japan). The medium was supplemented with 10% fetal bovine serum (FBS) (Thermo Fisher Scientific, MA, USA) and 1% antibiotic-antimycotic (Thermo Fisher Scientific). The culture plates were incubated at 37°C in a humidified atmosphere with 5% CO_2_. For TNF-α stimulation and exosome isolation, 50% confluent cell dishes were washed twice with 5 mL phosphate-buffered saline (PBS) (Nacalai tesque, Kyoto, Japan) and then incubated for 48 h in a medium supplemented with exosome-depleted FBS (System Biosciences, CA, USA) and with an antibiotic-antimycotic in the presence or absence of 1 ng/mL recombinant human TNF-α (Peprotech, NJ, USA).

### IL-6 quantification using ELISA

We quantified the amount of IL-6 secreted from MH7A cells by using Quantikine enzyme-linked immunosorbent assay (ELISA) human IL-6 (R&D Systems, MN, USA) according to the manufacturer’s instructions. Three independent experiments were performed. The cells remaining in each dish were treated with trypsin (Nacalai tesque) and counted thrice by using a hemocytometer (Waken B Tech, Kyoto, Japan). The amount of IL-6 was measured twice by using a VMax® Kinetic Microplate Reader (Molecular Devices, CA, USA) set to 490 nm with 564 nm wavelength correction. The standard curve was fitted with a four-parameter logistic curve by using ImageJ [32], and the results were then calculated. Statistical significance was determined by Welch’s t-test.

### Exosome isolation from MH7A-conditioned media

We isolated exosomes by ultracentrifugation from 200 mL of MH7A-conditioned media with or without TNF-α treatment, as described previously [33]. The conditioned media were collected and centrifuged at 2000 × *g* for 10 min at 4 ^°^C to eliminate dead cells, followed by filtration of the supernatant through a 0.2 μm filter unit (Thermo Fisher Scientific) to eliminate cell debris. The filtrates were stored at 4 ^°^C until ultracentrifugation. Exosomes were pelleted by ultracentrifugation at 100,000 × *g* for 160 min at 4 ^°^C (SRP28SA1, Hitachi Koki, Tokyo, Japan). The exosome pellets were washed once by resuspending them in PBS and ultracentrifugation. The pellets were again resuspended in 50 μL of PBS, rapidly frozen in liquid nitrogen, and stored at −80 ^°^C until use.

### Preparation of cellular protein

The MH7A-conditioned media were collected, and the cells were washed twice with 5 mL of ice-cold PBS, followed by the addition of 1 mL of radioimmunoprecipitation assay buffer (Wako) supplemented with 10 μL of a protease inhibitor cocktail (Sigma Aldrich, MO, USA). The cells were scraped off by a cell scraper and transferred to a microtube. The cell suspensions were kept on ice for 30 min with a vortex every 10 min. Cell debris was eliminated by centrifugation at 14,000 × *g* for 15 min at 4 ^°^C, and the supernatant was collected.

### Detection of protein by Western blot analysis

To quantify the protein concentrations of exosomes and cellular lysates, we used bicinchoninic acid assay (Protein Assay Bicinchoninate Kit, Nacalai tesque) according to the manufacturer’s instructions. In a reducing condition for the detection of HSP70 [34], cellular lysates and exosome samples (1.5–2.0 μg) were mixed with a sample buffer solution with 2-mercaptoethanol for sodium dodecyl sulfate polyacrylamide gel electrophoresis (Nacalai tesque) and then heated for 3 min at 98 ^°^C. In a nonreducing condition for the detection of CD63 and CD81 [34], cellular lysates and exosome samples were mixed with a sample buffer solution without 2-mercaptoethanol (Nacalai tesque) and incubated for 30 min at 37 ^°^C. The samples were then electrophoresed on in-house 15% polyacrylamide gels and blotted on nitrocellulose membranes (Bio-Rad, CA, USA). The membranes were blocked for 1 h in Tris-buffered saline with 5% skim milk (Wako) and 0.05% Tween-20 (Nacalai tesque). Thereafter, they were incubated with monoclonal antibodies against CD63 (#Ts63, Thermo Fisher Scientific), CD81 (#1.3.3.22, Santa Cruz Biotechnology, CA, USA), or HSP70 (#MAB1663, R&D Systems); horseradish peroxidase-conjugated anti-mouse antibody (Bio-Rad) was used as a secondary antibody. Immunoreactive bands were detected using Chemi-Lumi One Super (Nacalai tesque) and Image Quant LAS 4000 mini (GE Healthcare, IL, USA).

### RNA extraction

Exosomal RNAs and total cellular RNAs were isolated using the miRNeasy mini kit (QIAGEN, Hilden, Germany) according to the manufacturer’s instructions. First, carrier RNA was synthesized using Greiner Bio-One (Kremsmünster, Austria) (sequence: MS2 bacteriophage RNA [961–1000 bases] 5’-UCGCGACGUAUCGUGAUAUGGUUUUACAUAAACGAUGCAC-3’). The carrier RNA sequence was confirmed to not match the human genome by using BLAST [35]. To isolate exosomal RNAs, 1 μg of carrier RNA was spiked-in lysates prepared by the QIAzol lysis reagent from the miRNeasy mini kit. The quantity, size, and purity of the isolated RNA samples were analyzed by using Agilent 2100 Bioanalyzer (Agilent Technologies, CA, USA) with Agilent RNA 6000 Pico Kit (Agilent Technologies) according to the manufacturer’s instructions. The cellular RNAs of RNA integrity number > 7 were used for library preparation and are described in the following.

### Library preparation and sequencing

cDNA libraries were prepared using NEBNext® Multiplex Small RNA Library Prep Set for Illumina® (Set 1) (New England Biolabs, MA, USA) according to the manufacturer’s instructions with modifications on library size selection. Amplified cDNA libraries were electrophoresed on 12.5% polyacrylamide gel (E-T 12.5L, ATTO, Tokyo, Japan), and ~140 nucleotide bands (the length of adapter-ligated miRNA constructs) were eluted and precipitated by ethanol. The library sizes were checked by a bioanalyzer using the Agilent High Sensitivity DNA Kit (Agilent Technologies). The libraries were quantified by using a 7500 real-time polymerase chain reaction system (Applied Biosystems, CA, USA) with KAPA Library Quantification Kits (Kapa Biosystems, MA, USA) according to the manufacturer’s instructions. Furthermore, the libraries were equimolarly pooled for sequencing. Thereafter, sequencing was performed using MiSeq (Illumina, CA, USA) with single-end 36 cycles and 10% PhiX control (Illumina). The raw sequencing data are available from the database of the National Center for Biotechnology Information (SRA accession: SRP136572).

### Data analysis

**Fig 2:**
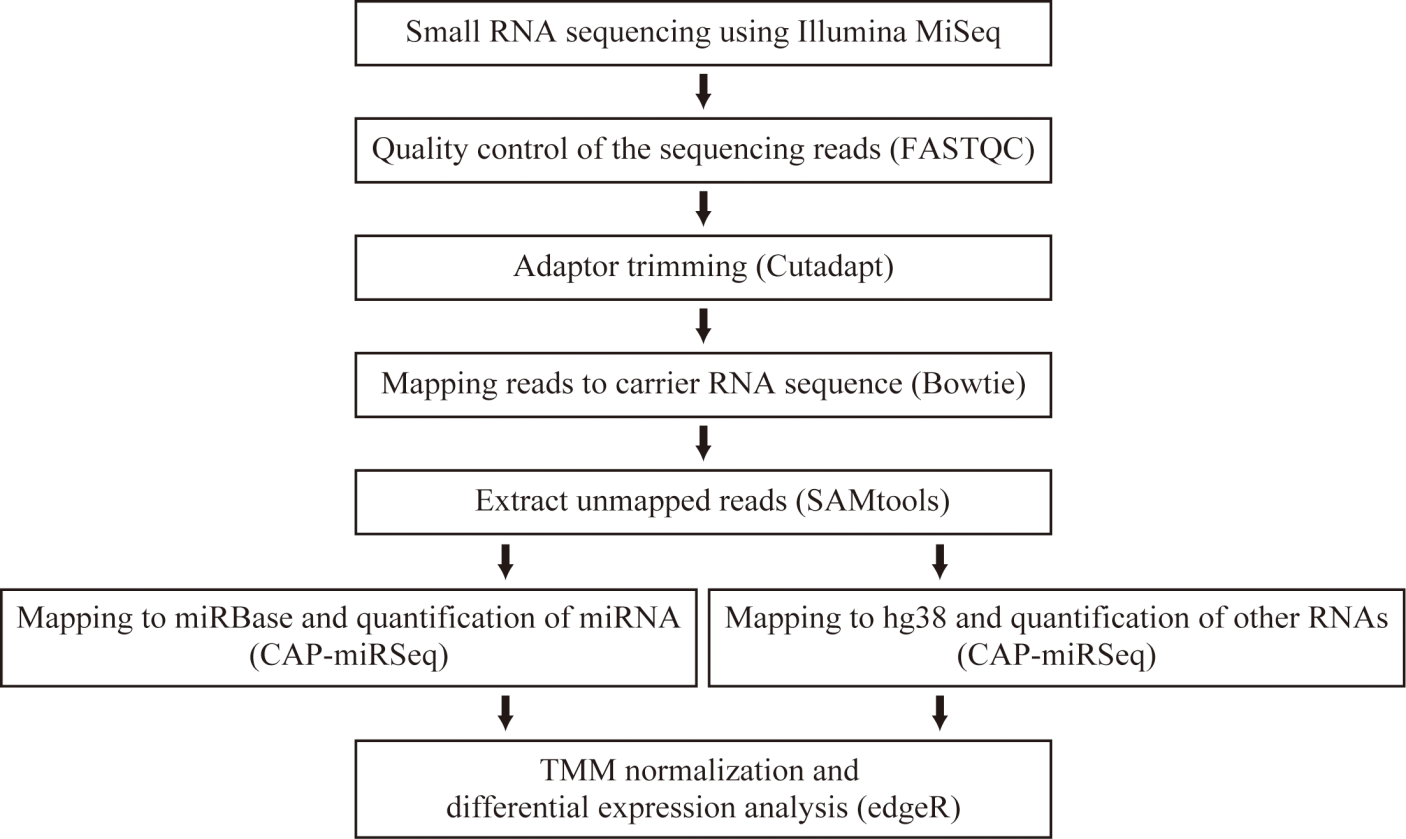
Flowchart of data analysis. After a quality check and adaptor trimming, the elimination of carrier RNA-Seq reads was performed using these bioinformatics tools. Thereafter, CAP-miRSeq was used to perform mapping and read counts. Differential expression analysis was conducted with edgeR. The software programs used for analysis are shown in parentheses.

Fig 2 shows a flowchart of the data analysis conducted in this study. First, we assessed the quality of raw sequencing data by using FastQC (version 0.10.1 downloaded from https://www.bioinformatics.babraham.ac.uk/projects/fastqc/). Thereafter, we preprocessed raw sequence reads by using cutadapt (version 0.9.5) [36] to trim the adaptor sequences and remove reads that were too short (< 17 base pairs in length). To remove carrier RNA sequence reads from exosome libraries, adapter-trimmed reads were aligned to the carrier RNA sequence by using Bowtie (version 0.12.7) [37]. Unmapped to the carrier RNA sequences were extracted from the SAM file created and were converted to FASTQ format by using SAMtools (version 0.1.19) [38]. We then performed mapping against human genome GRCh38/hg38 and a known miRNA database (miRBase release 21) [18] by using CAP-miRSEQ (version 1.1) [39] with default parameters, which output the read counts of RNA categories in Ensembl 83 [40] and mature miRNA expression counts. The read counts of each RNA category were normalized with the number of reads per genome-aligned reads, and the average total number of each RNA category was calculated. The trimmed mean of M-values (TMM) normalization [41] and differential expression analysis were performed using an R package called edgeR (version 3.16.5) [42, 43] via the quantile-adjusted conditional maximum likelihood method [44]. For TMM normalization, lowly expressed transcripts (showing < 5 counts in four or more libraries) were filtered out. The differential expression of transcripts other than miRNAs was performed using genome-aligned reads without miRNA-aligned counts. Transcripts with a false discovery rate (FDR) below a threshold of 0.05 determined by edgeR were considered differentially expressed.

### Target prediction of miRNAs

The target prediction of miRNAs was performed using DIANA-microT-CDS [45, 46] and TargetScan [47]. The DIANA-microT-CDS threshold was set to 0.7 (default). The result obtained using DIANA-microT-CDS included information about whether TargetScan also predicted the same genes. Only genes that were predicted by both algorithms were retrieved.

## Results

### Stimulation of MH7A cells by TNF-α

**Fig 3:**
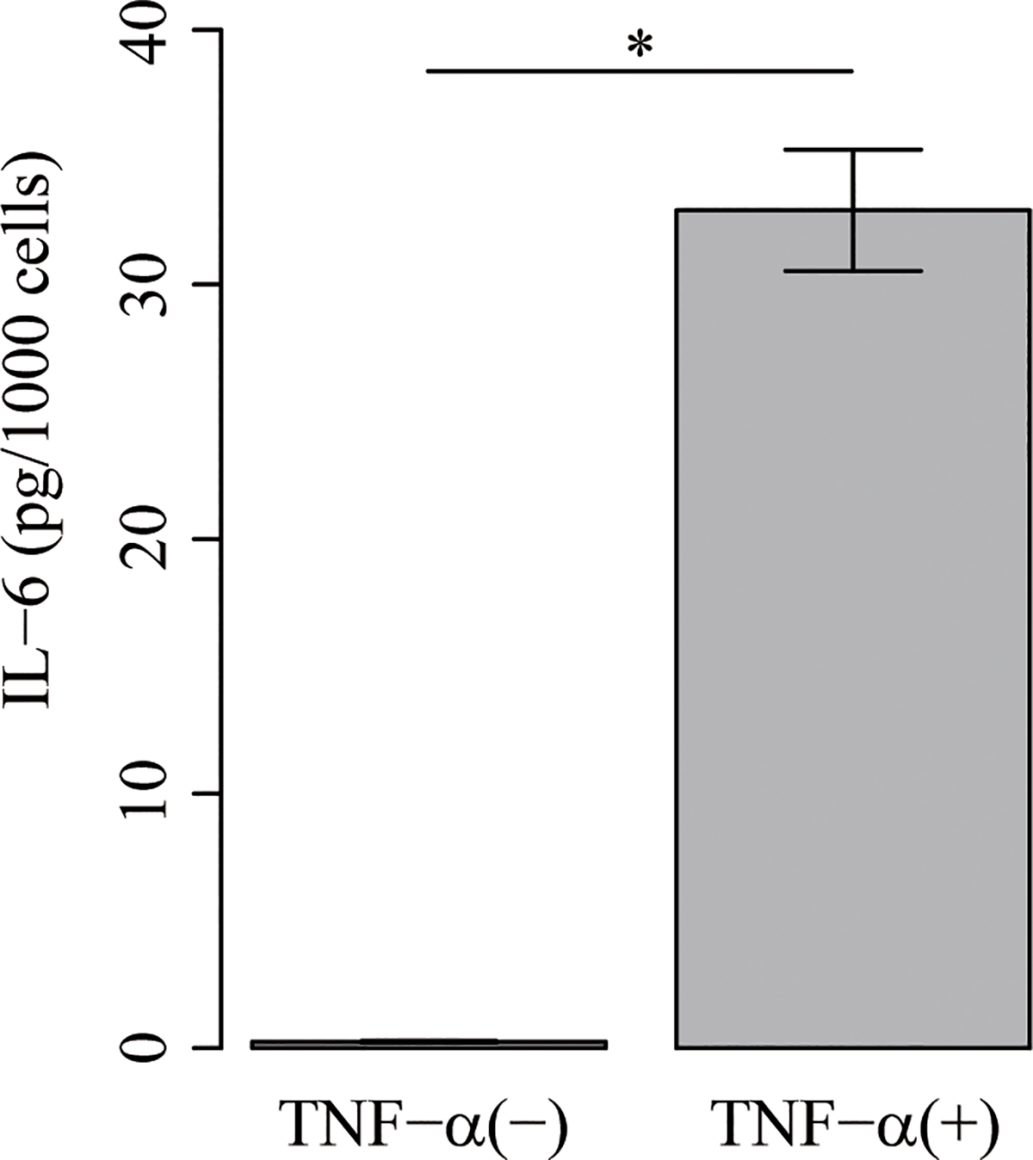
IL-6 secretion with or without TNF-α stimulation. Values are given as mean ± standard error (*n* = 3). *Statistical significance was determined by Welch’s t-test (*P* < 0.01).

We cultured MH7A cells obtained from RA patients with or without TNF-α for 48 h. MH7A cells respond to stimulation by nuclear factor kappa B (NF-κB) activator, TNF-α, and IL-1β to secrete cytokines such as IL-6 [31, 48]. MH7A responsiveness was quantified by measuring the IL-6 concentration of the MH7A-conditioned media. We found that the IL-6 secretion of MH7A significantly increases by TNF-α stimulation: TNF-α (+), 32.9 ± 2.4 pg/1000 cells; TNF-α (−), 0.245 ± 0.031 pg/1000 cells (see Fig 3). The results demonstrated that MH7A cells exhibit inflammatory response in our experimental conditions.

### Assessment of exosomes derived from MH7A cells

**Fig 4:**
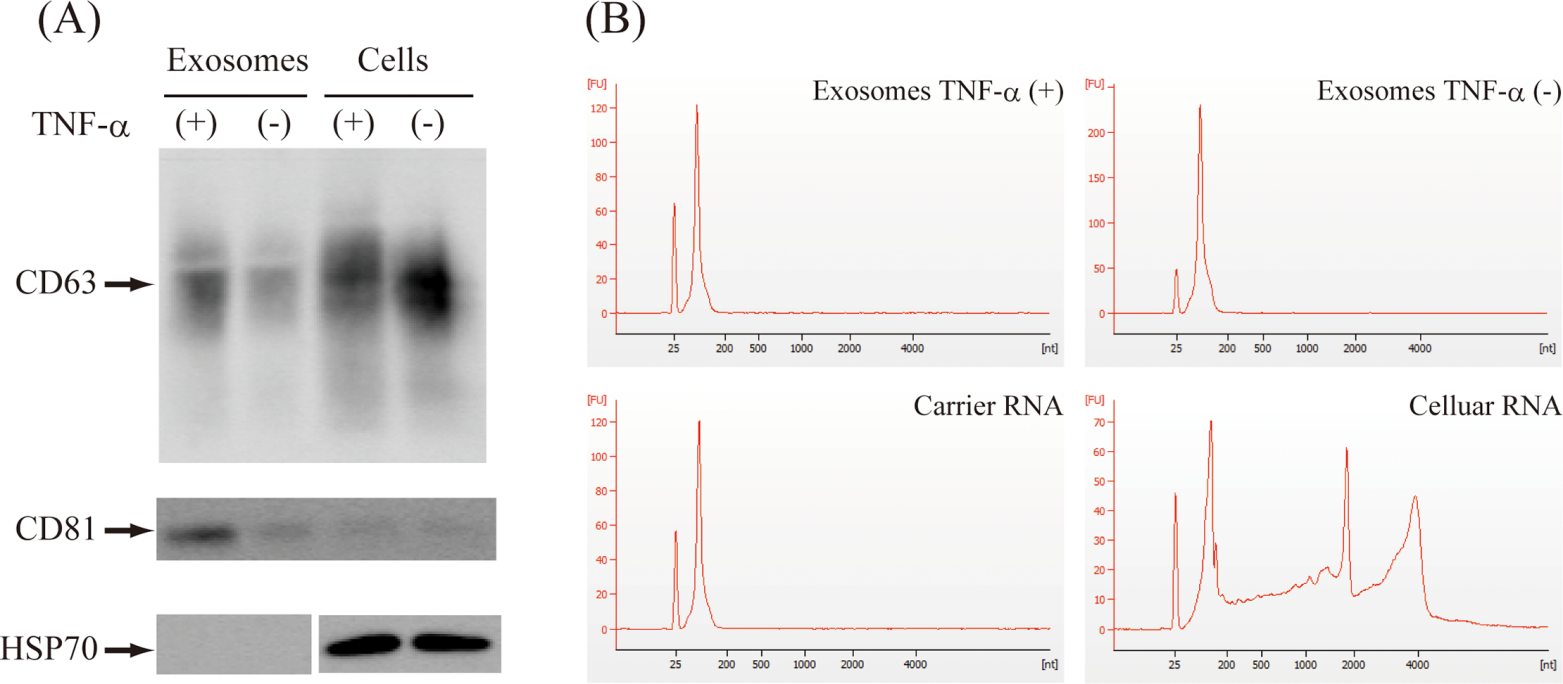
Assessment of exosomes derived from MH7A cells. (A) The Western blot analysis of exosome markers. (B) The size distribution of RNA molecules extracted from exosomes with or without TNF-α stimulation. Carrier RNA was also analyzed as a control. The cellular RNAs of MH7A were loaded as a positive control for rRNAs. The vertical axis shows fluorescent units (FU), and the scales were individually set. The horizontal axis shows electrophoretic mobility (RNA length). Lower marker peaks appeared at 25 nt. FU, fluorescent units. nt, nucleotides.

Samples were isolated from MH7A-conditioned media (MH7A cells were cultured with or without TNF-α stimulation). To detect exosomes in the isolated samples, we analyzed exosomal marker proteins (CD63, CD81, and HSP70 with Western blot analysis (Fig 4A). We found CD63 enrichment and detected CD81 in the samples. However, we did not find HSP70. In total cellular lysates, CD63 and HSP70 production was observed. Previous studies have shown that exosomal markers, including HSP70, are produced variably in exosomes derived from various cells [49]. This variability explains the nondetectable levels of HSP70 in MH7A exosomes. RNAs extracted from exosomes with synthesized carrier RNA (MS2 bacteriophage RNA) were analyzed with an Agilent Bioanalyzer RNA 6000 Pico chip. We found no rRNA peaks (two distinct peaks in the >1500 nt region), and cellular RNAs were not contaminated in the exosome samples with or without TNF-α stimulation (Fig 4B). In this study, we added carrier RNA to extract very small amount of exosomal RNAs. The amount of carrier RNA account for the majority, therefore, the peak profiles were very similar to those of the sample containing only carrier RNA.

### Sequencing of exosomal and cellular RNAs derived from MH7A cells

**Fig 5:**
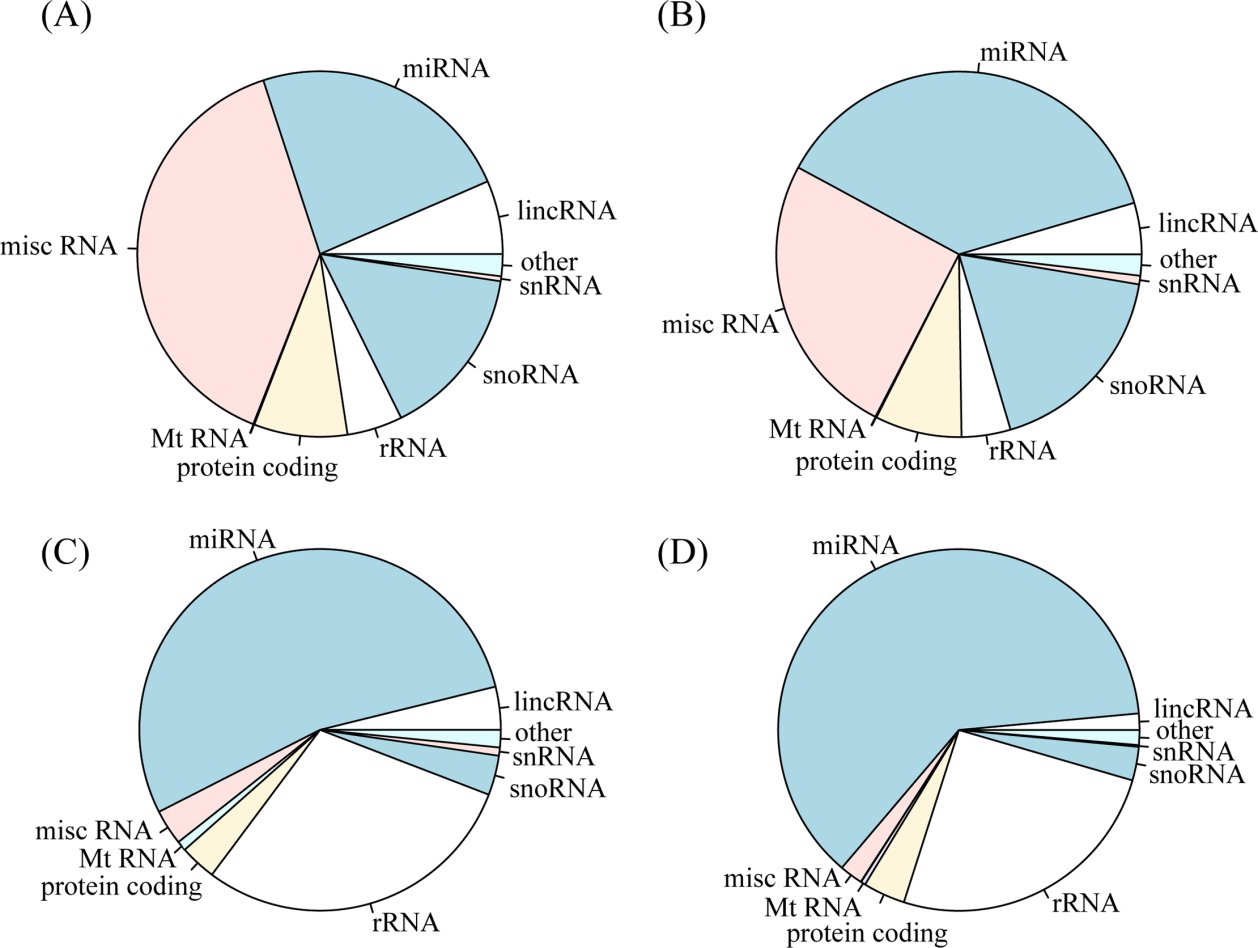
Distribution of RNA species. Exosomal RNAs with (A) or without (B) TNF-α stimulation. Cellular RNAs with (C) or without (D) TNF-α stimulation. lincRNA, long intergenic noncoding RNA; snRNA, small nuclear RNA; snoRNA, small nucleolar RNA; mtRNA, mitochondrial RNA; misc RNA, miscellaneous RNA.

The small RNA-Seq of exosomal and cellular RNAs of MH7A cells was performed with Illumina MiSeq. Spiked-in carrier RNAs were sequenced, along with exosomal RNAs; thus, we eliminated the sequence reads of carrier RNAs from exosome sample libraries by mapping to the carrier RNA sequence (5’-UCGCGACGUAUCGUGAUAUGGUUUUACAUAAACGAUGCAC-3’) by using Bowtie (version 0.12.7). The adaptor-ligated carrier RNA library was sequenced as a control. Exosomal and cellular RNAs with or without TNF-α stimulation were sequenced three times independently; thus, we sequenced 13 sequencing libraries and generated 66,043,334 reads (S1 Table). After preprocessing and eliminating carrier RNA sequence reads for exosome libraries, we found that 1,565,748 and 19,843,207 reads were aligned to the human genome in exosome libraries and cell libraries, respectively (S1 Table). Diverse categories of RNAs annotated by Ensembl were detected similar to those in exosomal RNA libraries, whereas miRNAs comprised a large proportion in cellular RNA samples (see Fig 5 and S1 File). Table 1 shows the expressions of transcripts belonging to RNAs other than miRNAs. Among these, the most abundant transcript was one of Y RNAs, RNY4 [19], which was not significantly induced with TNF-α stimulation (Table 1).

**Table 1:** Top 10 most abundant transcripts other than miRNAs in exosomes.

Gene name	Ensemble ID	Gene type	Log2 FC	Log2 CPM	P Value	FDR
RNY4	ENSG00000252316.1	misc RNA	0.857	16.6	0.552	0.9
RNY4P10	ENSG00000202441.2	misc RNA	0.856	16.6	0.552	0.9
RNY4P7	ENSG00000201470.1	misc RNA	0.858	16.5	0.548	0.9
RNY4P20	ENSG00000252487.1	misc RNA	0.847	16.5	0.546	0.9
SNORD3A	ENSG00000263934.4	snoRNA	−0.0875	15.8	0.943	0.987
SNORD3C	ENSG00000264940.4	snoRNA	−0.0878	15.8	0.942	0.987
SNORD3B-2	ENSG00000262074.6	snoRNA	−0.0895	15.8	0.941	0.987
SNORD3B-1	ENSG00000265185.5	snoRNA	−0.0895	15.8	0.941	0.987
RNY1	ENSG00000201098.1	misc RNA	0.467	15.8	0.725	0.947
VTRNA1-1	ENSG00000199990.1	misc RNA	0.254	14.2	0.0654	0.798

FC, fold change; CPM, counts per million; FDR, false discovery rate; misc RNA, miscellaneous RNA; snoRNA, small nucleolar RNA.

The number of reads mapped to the mature miRNA sequences in miRBase (release 21) were 485,507 and 12,747,590 in exosome libraries and cell libraries, respectively (S1 Table and S2 File). On average, we detected 223 and 663 known miRNAs with ≥5 coverage in exosome libraries and cell libraries, respectively. In the adaptor-ligated carrier RNA library, only 340 reads were aligned to the mature miRNA sequence in miRBase (release 21) (S1 Table and S2 File). Among these 340 reads, most were aligned to miR-21-5p (122 reads) and miR-100-5p (41 reads), which were abundant in other libraries (S2 Table and S2 File). This may be explained either by cross-contamination between samples during library precipitation or by the false assignment of index sequences to the samples in Illumina MiSeq sequencing (also known as index hopping) [50]. The number of miRNA reads detected in the adaptor-ligated carrier RNA library was extremely small compared with that of other libraries; hence, we concluded that the use of carrier RNA would not affect exosomal RNA-Seq.

**Fig 6:**
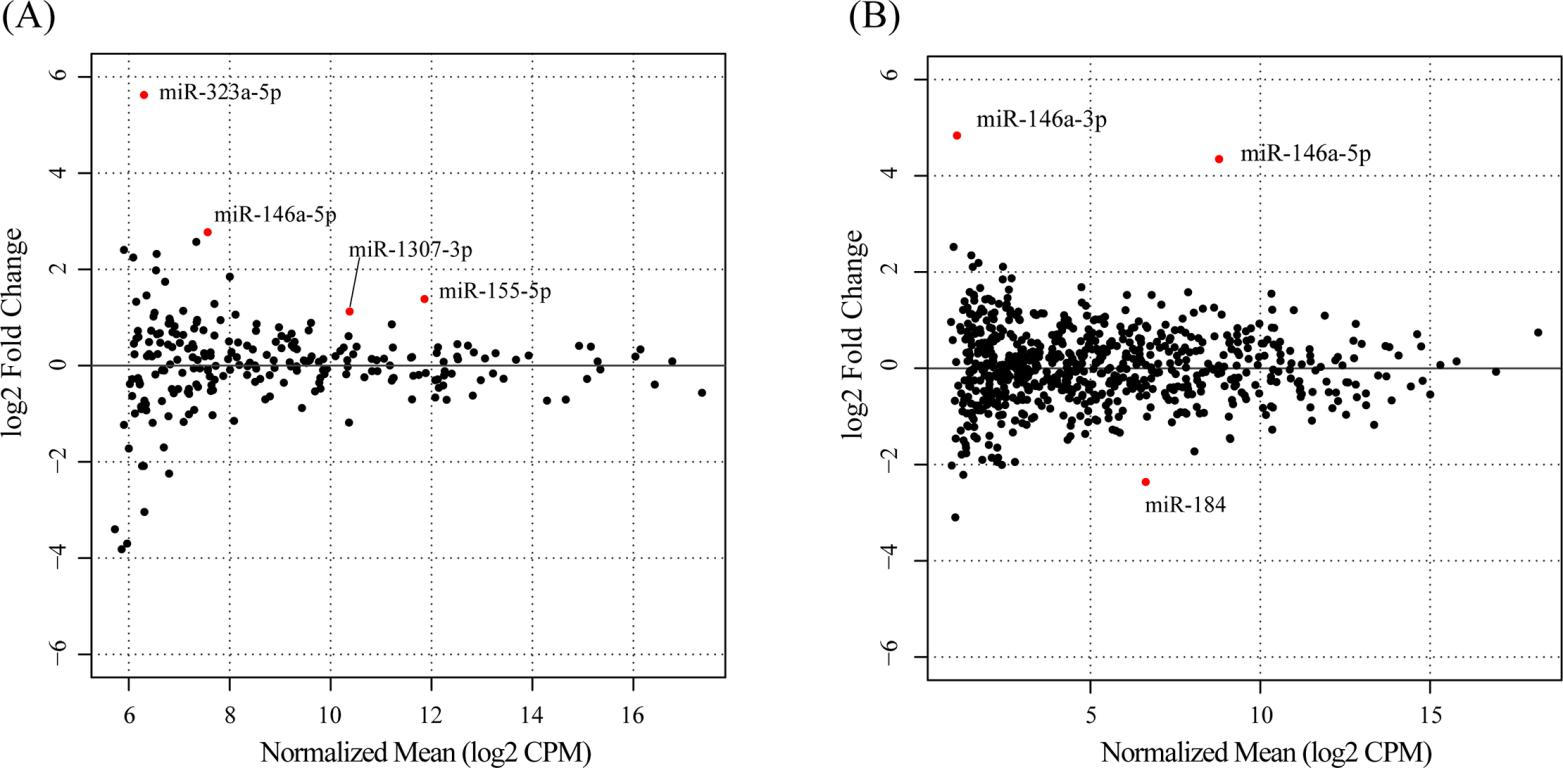
MA plot (stimulated vs. nonstimulated) of miRNAs. Exosomal RNAs (A) and cellular RNAs (B). Each point represents one miRNA. miRNAs upregulated with TNF-α stimulation are above the center horizontal line, and miRNAs downregulated with TNF-α stimulation are below the center horizontal line. miRNAs with FDR < 0.05 are shown as red dots. CPM, counts per million.

Among the exosomal miRNAs detected, four miRNAs (miR-155-5p, miR-146a-5p, miR-323a-5p, and miR-1307-3p) were significantly upregulated by TNF-α stimulation (FDR < 0.05) (red dots in Fig 6A, Table 2, and S3 File); significantly downregulated miRNAs were not detected in the exosomes. Among the cellular miRNAs detected, three miRNAs (miR-146a-5p, miR-184, and miR-146a-3p) were differentially expressed by TNF-α stimulation (FDR < 0.05) (red dots in Fig 6B, Table 2, and S3 File). miR-146a-5p and miR-146a-3p were significantly upregulated, whereas miR-184 was downregulated. By comparing the expression changes between exosomes and cells, we found that the expression variations of miR-323a-5p and miR-1307-3p were less than two times in the cells even though miR-155-5p and miR-146a-5p expressions were upregulated more than twice in both exosomes and cells (Table 3). These observations suggested that MH7A cells with TNF-α stimulation may preferentially load miR-323a-5p and miR-1307-3p onto exosomes (Table 3).

**Table 2:** Differentially expressed miRNAs.

	miRNA	Log_2_ FC	Log_2_ CPM	*P* Value	FDR
Exosomes	miR-155-5p	1.38	11.9	0.0000191	0.00456
	miR-146a-5p	2.77	7.56	0.000145	0.0174
	miR-323a-5p	5.62	6.30	0.000388	0.0268
	miR-1307-3p	1.13	10.4	0.000448	0.0268
Cells	miR-146a-5p	3.57	9.41	4.70×10^−14^	3.34×10^−11^
	miR-184	−2.36	6.63	8.55×10^−6^	0.00304
	miR-146a-3p	4.02	2.17	6.71×10^−5^	0.0159

FC, fold change; CPM, counts per million; FDR, false discovery rate.

**Table 3:** Comparison of expression variation of four miRNAs significantly upregulated in exosomes.

miRNA	Log_2_ FC in cells	Log_2_ FC in exosomes
miR-155-5p	1.09	1.38
miR-146a-5p	3.56	2.77
miR-323a-5p	0.261	5.62
miR-1307-3p	−0.269	1.13

FC, fold change.

Among the four miRNAs differentially expressed in exosomes, miR-323a-5p and miR-1307-3p were first annotated as possible candidates for RA-related miRNAs. By using DIANA-microT-CDS and TargetScan, we obtained 336 and 20 predicted target genes of miR-323a-5p and miR-1307-3p, respectively (S3 Table). The miR-323a-5p-predicted target genes included CD6 which is produced on the T-cell plasma membrane [51], whereas the miR-1307-3p-predicted target genes included *NDRG2* which reportedly affects osteoclast differentiation [52]. These predicted genes may be involved in RA pathogenesis.

## Discussion

In this study, we quantified RASF exosomal RNAs with TNF-α stimulation. This is the first study to comprehensively analyze RASF-derived exosomal miRNAs. We found that four miRNAs (miR-155-5p, miR-146a-5p, miR-323a-5p, and miR-1307-3p) were upregulated in RASF exosomes by TNF-α stimulation (as shown in Fig 6A and Table 2). The selective loading of miRNA into exosomes have been reported [13, 53, 54]. For example, nSMase2 regulated the number of exosomal miRNAs [55]. Sumoylated hnRNPA2B1 recognizes 3’ motif of RNAs and selectively packed them into exosomes [56]. Furthermore AGO2 was identified in exosomal proteins [57], and knockout of AGO2 could decrease the preferentially-loaded miRNAs [58]. However, there are still ambiguity in TNF-α-dependent or–independent mechanisms, and they remain to be clarified.

Our sequencing data indicated that MH7A exosomes contain various types of small RNAs annotated in Ensembl, such as snoRNAs, lincRNA, and misc RNA, as well as miRNAs. This result is consistent with previous reports that exosomes contain various types of RNAs [59]. In particular, we found that misc RNAs are conspicuously expressed in exosomes, along with miRNAs. In the misc RNAs identified in our study, Y RNAs were included. This is consistent with previous studies showing the existence of Y RNAs in exosomes [59]. hY4 (gene names annotated as RNY4), one of the Y RNAs abundantly present in MH7A exosomes (shown in Table 1), is suggested as a signaling driver of Toll-like receptor 7 in monocytes [29]. Therefore, although hY4 was not differentially expressed with TNF-α stimulation in the present study (Table 1), abundant exosomal hY4 might contribute to an inflammatory positive-feedback loop in RA-affected joints. However, further studies are required to clarify the function of extracellular Y RNAs and the loading mechanism of these RNAs.

It is noteworthy that miR-155-5p and miR-146a-5p, which are reportedly involved in RA pathogenesis [60], were expressed in exosomes derived from the RASF cell line MH7A and upregulated with TNF-α stimulation. A previous study reported that miR-155-5p and miR-146a-5p are upregulated in the synovial fluid in RA compared with osteoarthritis (OA) [22]. This suggested that our experimental model is valid. With RASF-aberrant growth and abundance in the synovium in RA [2], upregulated miR-155-5p and miR-146a-5p in RASF exosomes appear to contribute to an increase in these miRNAs in the synovial fluid in RA.

miR-155-5p is reportedly involved in RA pathogenesis and highly expressed in synovial fibroblasts [23, 61], synovial tissues [23], peripheral blood mononuclear cells (PBMCs) [61], and macrophages [62] in RA patients compared with healthy controls or OA patients. miR-155-5p also regulates inflammation response and the development of each lymphocyte subset, such as B-cells [63], CD8+ [64], and CD4+ T-cells [65]. miR-155-deficient mice do not develop collagen-induced arthritis (an RA model in mice); hence, miR-155 has been implicated as a crucial miRNA in RA pathogenesis [24]. On the other hand, miR-155-5p is confirmed to suppress production of matrix metalloproteinase-3 production via the downregulation of the inhibitor of NF-κB kinase subunit epsilon, thus suggesting that miR-155-5p is a protective factor in joint destruction [61]. Therefore, the intercellular transportation of miR-155-5p derived from RASF exosomes might play a dual role in the development of arthritis and protection from joint destruction.

The roles of miR-146a-5p in RA seem confusing [21, 60]. miR-146a-5p expression is reportedly upregulated by TNF-α stimulation in vitro [23, 66] and increases in synovial tissue [66], synovial fibroblasts [23], CD4^+^ T-cells [67], PBMCs [68], synovial fluid [22], and plasma [22] in RA patients compared with healthy controls. Studies have confirmed that miR-146a-5p targets TNF receptor associated factor 6 (TRAF-6) and interleukin-1 receptor associated kinase 1 (IRAK-1) in vitro [69]. However, despite increased miR-146a-5p expression, TRAF-6 and IRAK-1 production did not change significantly in PBMCs obtained from RA patients compared with healthy controls [68]. Furthermore, miR-146a-5p overexpression suppressed apoptosis in Jurkat cells used as a T-cell model, thus indicating that miR-146-5p is an inflammatory agent [67]. On the other hand, the administration of double-stranded miR-146a-5p prevented joint destruction in a collagen-induced arthritis mouse model [70]. Considering that miR-146a-5p can be a positive or negative regulator of inflammation and joint destruction, our observation that miR-146a-5p is detected and upregulated with TNF-α stimulation in RASF exosomes would help us determine the role of miR-146a-5p in RA pathogenesis.

We observed that miR-323a-5p and miR-1307-3p, as well as miR-155-5p and miR-146a-5p, were upregulated with TNF-α stimulation in exosomes. miR-323a-5p and miR-1307-3p were first shown to be relevant in RA, and further studies are required to detect these miRNAs *in vivo*. Among the predicted target genes, we included those whose functions are relevant to inflammation and joint destruction: miR-323a-5p can target CD6, and miR-1307-3p can target *NDRG2* (S3 Table). CD6 is an outer membrane protein of T-cells [71]. It was formerly regarded a costimulatory receptor of T-lymphocytes [72, 73] but has been revealed to attenuate the signaling of T-cell activation [74]. Therefore, miR-323a-5p transport to T-cells has the potential to reinforce synovium inflammation in RA. The overexpression of *NDRG2*, one of the predicted target genes of miR-1307-3p, has been shown to inhibit osteoclast differentiation from monocytes [52]. Furthermore, NDRG2 inhibits the expression of osteoclast-related genes, including the receptor activator of NF-κB (RANK) in monocytes [52]. By contributing to osteoclastogenesis [75] in a coordinated manner with RANK ligand expression, the exosomal miR-1307-3p transported to monocytes has the potential to promote osteoclast differentiation by targeting *NDRG2* and cause joint destruction.

We found that in our sequencing data, exosomal and intracellular miRNA expression with TNF-α stimulation was different among miR-155-5p, miR-146a-5p, miR-323a-5p, and miR-1307-3p. miR-146a-5p was significantly upregulated in both exosomes and cells, and miR-155-5p was upregulated by twofold in both. By contrast, miR-323a-5p and miR-1307-3p were significantly upregulated only in exosomes, and the intracellular expression variation between them was less than twofold. Studies have reported that a subset of miRNAs is preferentially sorted from the cytosol to exosomes [27, 76–78]. Thus, MH7A cells with TNF-α stimulation may preferentially load miR-323a-5p and miR-1307-3p to exosomes.

The role of exosomes in RA pathogenesis has been investigated earlier but remains unclear [79]. Exosomes contain numerous bioactive molecules, such as proteins, mRNAs, miRNAs, and other RNAs. The physiological activity of exosomes reflects the multiple effects of their molecular cargo. In the case of RASF exosomes, T-cell resistance to apoptosis is neutralized only partly by soluble TNF receptor-1, thus indicating that exosomal molecular cargo other than TNF-α contributes to T-cell apoptosis resistance [17]. Furthermore, the physiological activity of exosomes differs among recipient cell types. Our findings on RASF exosomal RNAs suggest that future studies using more physiological models are required to identify target cells and to explore the physiological activity of RASF exosomes.

In conclusion, our study quantified small RNAs in RASF exosomes. Differential expression analysis identified that four miRNAs are differentially expressed in exosomes with TNF-α stimulation. Our data on the RNA molecular cargo of RASF exosomes will help us in understanding the role of exosomes in RA pathogenesis.

## Supporting information

S1 Table Summary of 13 libraries. (XLSX)

S2 Table Top 10 abundant miRNAs in exosome and cell. CPM, counts per million. FC, fold change.
(XLSX)

S3 Table Predicted target genes of miR-1307-3p and miR-323a-5p. Prediction score threshold was set to 0.7 as default.
(XLSX)

S1 File Transcripts read counts of all libraries. (XLSX)

S2 File miRNA read counts of all libraries. (XLSX)

S3 File TMM normalization and differential expression of miRNAs. CPM, counts per million. FC, fold change. FDR, false discovery rate.
(XLSX)
